# The role of soluble CD40L in autoimmune diseases

**DOI:** 10.1016/j.jtauto.2025.100288

**Published:** 2025-04-19

**Authors:** Meryem Mabrouk, Hicham Wahnou, Yahye Merhi, Haissam Abou-Saleh, Fadila Guessous, Younes Zaid

**Affiliations:** a Materials, Nanotechnologies and Environment Laboratory, Department of Biology, Faculty of Sciences, Mohammed V University in Rabat, Rabat, Morocco; b Immunology and Biodiversity Laboratory, Department of Biology, Ain Chock Faculty of Sciences, Hassan II University, Casablanca, Morocco; c Research Center, Laboratory of Thrombosis and Hemostasis, Montreal Heart Institute, Faculty of Medicine, Université de Montréal, Montreal, QC, H3T 1J4, Canada; d Biomedical Sciences Department, College of Health Sciences, Qatar University, Doha P.O. Box 2713, Qatar; e Oncopathology, Biology and Environment of Cancer Laboratory, Mohammed VI Center for Research and Innovation, Rabat, Morocco; f Faculty of Medicine, Mohammed VI University of Sciences and Health, Casablanca, Morocco; g Department of Microbiology, Immunology and Cancer Biology, School of Medicine, University of Virginia, Charlottesville, VA, USA

**Keywords:** CD40L, CD40, T lymphocytes, Platelets, Autoimmune disease

## Abstract

CD40^−^CD40L is essential for immune system modulation because it coordinates both adaptive and inflammatory responses.

Systemic lupus erythematosus, multiple sclerosis, inflammatory bowel disease, thrombocytopenic purpura, and rheumatoid arthritis are among the autoimmune illnesses in which it is especially prominent. Thus, the CD40^−^CD40L axis is a significant therapeutic target, despite the fact that its inhibition was first constrained by thromboembolic adverse effects.

New therapeutic approaches, such as nanotechnological methods and new-generation monoclonal antibodies, have been developed as a result of recent research with the goal of enhancing therapy efficacy and safety. This study opens up new avenues for the treatment of autoimmune illnesses by examining the pathophysiological consequences of CD40^−^CD40L and reviewing new treatments that target this pathway.

## Graphical abstract

**Figure:**
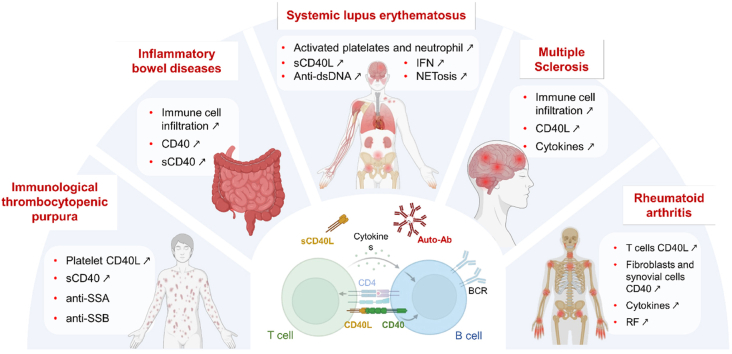

## 1 Introduction

**Fig. 1:**
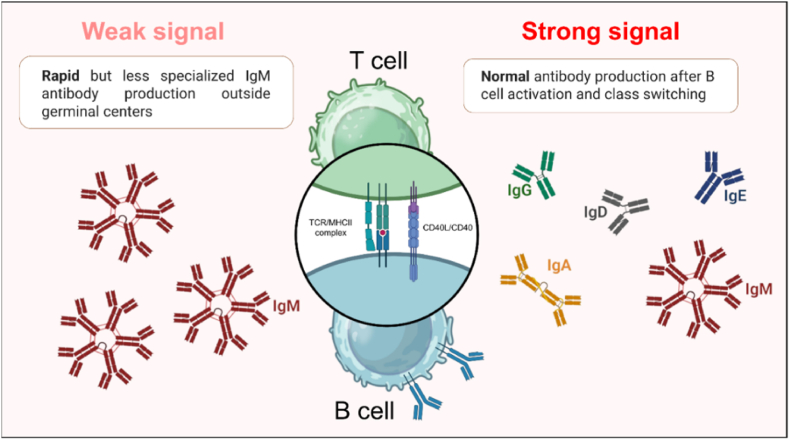
Graphical representation of the importance of the CD40^−^CD40L signal strength in lymphoblast differentiation and antibody production.

CD40 ligand (CD40L), also known as CD154, is a transmembrane glycoprotein with a trimeric structure belonging to the TNF (tumor necrosis factor) family [1]. It was first described on the surface of activated CD4^+^ T lymphocytes, interacting with CD40 on B lymphocytes. CD40 expression is constitutive in many immune cells (B lymphocytes, dendritic cells, monocytes, macrophages, basophilic polynuclear cells) but also in non-immune cells (endothelial cells, smooth muscle cells, fibroblasts, synoviocytes, epithelial cells) [2] (Table 1). CD40L expression is inducible, mainly by activated T lymphocytes and activated platelets. Platelets are considered the first reservoir of CD40L; CD40L stored in a pre-formed state in α-granules is expressed at the membrane a few minutes to a few hours after platelet activation. It is subsequently cleaved by metalloproteinases and released in the form of soluble CD40L (sCD40L) which retains its trimeric conformation [[3], [4], [5]] (see Fig. 1).

**Table 1:** Cell types expressing CD40 and CD40L [1,10].

CD40	CD40L
B lymphocyte	Activated T lymphocyte
Monocyte	Platelet
Dendritic cell	NK cell
Basophilic Polynuclear Cell	B lymphocyte
Eosinophilic polynuclear cell	Basophilic Polynuclear Cell
Endothelial cell	Eosinophilic Polynuclear Cell
Fibroblast	Monocyte
Smooth muscle cell	Dendritic cell
Keratinocyte	Endothelial cell
Platelet	
Neuron

Both membrane-bound and soluble forms of CD40L appear to retain the ability to form trimers to bind CD40 and enable biological signal transduction [[6], [7], [8]].

CD40^−^CD40L signal transduction involves associated adaptor proteins TRAF (TNF-receptor associated factor) or JAK (Janus Kinase) in the cytoplasmic domain of CD40, responsible for activating various pathophysiological pathways and involving second messengers such as MAPKs (mitogen-activated protein kinases), PI3K (phospho-inositide-3 kinase), PLCγ (phospholipase Cγ2) or STAT5 (signal transducer and activator of transcription 5). This transduction is responsible for the activation of NF-κB (nuclear factor kappa B), NFAT (nuclear factor of activated T-cells) and AP-1 (activator protein-1) and the transcription of genes encoding pro-inflammatory cytokines (IL(interleukin)-6, IL-10, TNFα), adhesion molecules (ICAM) or costimulation molecules (CD80/B7-1, CD86/B7-2) [6,9,10].

The CD40^−^CD40L axis plays a major role in autoimmune diseases [[11], [12], [13]]. Targeted therapeutics have been developed as a result of its role in the etiology of various disorders [14]. Despite their effectiveness, the early anti-CD40L monoclonal antibodies had thromboembolic adverse effects that limited their clinical application [15]. Significant developments in recent years have produced novel strategies such bispecific antibodies, nanoparticles, and unconventional inhibitors, as well as new generations of anti-CD40L antibodies that are tailored to prevent these side effects [16].Additionally, sCD40L is becoming a biomarker of interest, although a lack of standardization hinders its clinical application.

This review offers an up-to-date analysis of the role of CD40^−^CD40L in autoimmune diseases, exploring its physiological and pathological implications, as well as emerging therapeutic strategies aimed at targeting this pathway more effectively and safely. In particular, we highlight current challenges, new drug formulations and future prospects for the treatment of autoimmune pathologies associated with the CD40^−^CD40L axis.

## 2 Physiological implications of the CD40^−^CD40 ligand system

Specific recognition of an antigen by the B lymphocyte surface receptor (BCR) initiates the proliferation and differentiation of B lymphocytes into plasma cells, which synthesise and secrete antigen-specific immunoglobulins. This differentiation process also gives rise to memory cells, which are rapidly reactivated if the same antigen is encountered again. This succession of events describes the development of humoral immune response. This process requires cognate interactions between B lymphocytes and other immune system players such as T lymphocytes and DCs, commonly known as the immune synapse, which serves as a platform for the transfer of information from DCs towards T cells, leading to their activation [9]. Among the essential membrane contacts, the interaction between CD40 and CD40L is crucial at various levels [17].

The first stage of BL activation takes place in the T cell-rich regions of the secondary lymphoid organs, where there are also numerous interdigitating DCs expressing CD40 that have captured the antigen. Specific recognition by the TCR of the antigenic peptide presented by the MHC of DCs leads to the activation of T lymphocytes and in particular to the expression of CD40L, acting as a co-stimulatory molecule [17].

Recognition by the BCR on the surface of B lymphocytes of the antigen on the surface of DCs and the interaction of CD40 expressed by BLs with CD40L on the surface of activated T lymphocytes leads to the activation and proliferation of B lymphocytes. The extra-follicular reaction then gives rise either to lymphoblasts which initiate the germinal center reaction or to plasmablasts which differentiate into IgM-secreting plasma cells [17].

The lymphoblasts generated during the extra-follicular reaction proliferate intensely in the presence of follicular DCs and develop into centroblasts. During this stage, somatic mutations are randomly distributed throughout the variable regions of the Ig genes, resulting in the appearance of antibodies on the surface of the B lymphocytes with greater affinity for the antigen [17].

The centroblasts thus generated differentiate into non-proliferating centrocytes which form the clear zone of germinal center and are selected by the antigen presented by the follicular DCs. Subsequently, the high-affinity centrocytes selected are able to internalise the antigen and present it to the surrounding T lymphocytes, which are then activated, expressing CD40L on their surface. The engagement of CD40 on the surface of high-affinity centrocytes by CD40L on the surface of activated T lymphocytes enables them to survive. Self-reactive centrocytes are eliminated because they are unable to induce CD40L expression on the surface of T lymphocytes [17].

The centrocytes then undergo the isotypic switching process which takes place in the apical light zone. This critical step gives the humoral immune response its biological diversity, as each Ig subclass or isotype (IgM, IgG, IgA, IgE), defined by its specific Fc fragment, is responsible for different effector functions. The CD40 signal is necessary and mandatory for isotype switching. Interruption of the CD40/CD40L signal, as in hyper IgM syndrome, results in inhibition of the isotype switching process [18].

The sustained production of antibodies after the development of the immune response is ensured by the migration of plasmablasts from the germinal center to the bone marrow or to the lamina propria of the mucosa, where they can secrete large quantities of high-affinity immunoglobulins. Upon a second encounter with the antigen, the humoral response is provided by circulating memory B cells. Although the molecular signals that lead to the terminal differentiation of centrocytes into memory B cells or plasmablasts are still poorly understood, several teams have demonstrated the role of the CD40/CD40L pair in the decision to differentiate into memory cells [19].

The fact that CD40 is expressed not only by B lymphocytes but also by other antigen-presenting cells (APCs) suggests that the CD40^−^CD40L pair is involved in the initiation and effector phases of the cellular immune response. Various studies have shown the role of CD40/CD40L interactions in regulating APC activation and T lymphocyte priming [20].

Interactions between APCs and T lymphocytes are necessary for the initiation and unfolding of the cellular immune response. These interactions give rise to bi-directional activation signals which are important for the activation of antigen-specific T lymphocytes and for the activation of APCs. Two distinct signals are required for T cell activation [21]. The first signal consists of the formation of the ternary complex by the interaction of the TCR on the surface of the T lymphocyte with the MHC/antigenic peptide complex on the surface of the APC. The second signal is delivered by the interaction of co-stimulatory molecules such as CD80 and CD86 on the surface of APCs with CD28 on the surface of T lymphocytes.

Various studies have demonstrated that the interaction of CD40 on the surface of APCs with CD40L is an APC activation signal. It has been shown that the increased expression of the co-stimulatory molecules CD58, CD80 and CD86 by Langerhans cells was caused by the interaction of CD40 on their surface with CD40L expressed by cells transfected for this same molecule [22].

In addition, the interaction of CD40L with CD40 on the surface of dendritic cells regulates the production of certain pro-inflammatory cytokines such as IL-8, MIP-1α, TNF-α, IL-12 and IFN-α [23]. The production of these cytokines by DCs is fundamental for the induction of the inflammatory response; the secretion of IL-12 secondary to the engagement of CD40 on DCs is, for example, necessary for the activation of T lymphocytes and the development of a Th1-type response [24].

On the other hand, the interaction of CD40 with CD40L is indeed important for the engagement of naive CD4^+^ T lymphocytes in the adaptive immune response ('priming'). Studies carried out in mice invalidated for the CD40L gene confirm the importance of CD40/CD40L interactions for the priming of T lymphocytes against peptide antigens. For example, Grewal et al. showed that CD4^+^ T lymphocytes from CD40L-disabled mice immunised with a peptide antigen were unable to proliferate or produce IL-4 and IFN-γ *in vitro* in the presence of the same antigen [25].

Other authors have also shown that CD40/CD40L interactions are involved in the allogeneic cellular immune response by demonstrating that T lymphocytes from CD40L−/− mice, in contrast to T lymphocytes from wild-type mice, cannot cause graft-versus-host disease (GVHD) when transferred to transplanted mice [26].

More importantly, CD40/CD40L interactions are critical for the priming of CD8^+^ T lymphocytes during the cytotoxic response. Cross-priming involves APC presenting an exogenous antigen via MHC I to cytotoxic T lymphocyte (CTL). During cross-priming, the LT helper (LTh) and the CTL must recognise the antigen presented on the surface of the same APC. Interactions by soluble factors, particularly between the LTh and the APC, are necessary for the APC to ‘prime' the naive CTL.

Schoenberger has shown in mice “depleted” of CD4^+^ TL that an agonist anti-CD40 antibody alone can “prime” CTLs during immunisation with a peptide antigen [27]. This work therefore confirms the importance of the interaction of CD40 on the surface of APC with CD154 on the surface of hTLs for CTL priming.

The CD40/CD40L interaction is involved in the formation of platelet-leukocyte aggregates (PLA), in particular platelet-MC and platelet-neutrophil aggregates, which are linked to several inflammatory and thrombotic conditions such as cardiovascular disease, rheumatoid arthritis and inflammatory bowel disease [28]. Reduced PLA numbers have been detected with both CD40L−/− platelets and CD40−/− leukocytes [28]. At the site of inflammation, platelets adhering to the intravascular wall capture leukocytes through CD40/CD40L interaction, resulting in leukocyte infiltration [29].

CD40 signaling in macrophages results in a pro-inflammatory environment that may be beneficial for host defense, but detrimental in autoimmune or inflammatory diseases. For example, a recent study demonstrated that blocking TRAF6 signaling in human macrophages, while preserving CD40-TRAF2/3/5 signaling, was effective in reducing atherosclerosis while keeping CD40/CD40L intact for appropriate immune responses [30].

## 3 Pathological implications of the CD40^−^CD40 ligand system

The crucial role of the CD40/CD40L interaction *in vivo* was first described in hyper IgM syndrome, an X-linked immune deficiency caused by an alteration in the gene encoding CD40L. This syndrome is characterised by a severe deficiency in serum immunoglobulins IgG, IgA and IgE, contrasting with normal or elevated IgM and susceptibility to opportunistic infections. It illustrates the importance of the interaction between the B lymphocyte CD40 receptor and the T lymphocyte CD40L in generating germinal centers, the T-dependent humoral response and the generation of memory B lymphocytes, and demonstrates the absence of a compensatory mechanism shedding light on the intricate interplay between immune cell subsets and its implications in autoimmune pathology [12]. Transitioning to the link between the CD40/CD40L interaction and autoimmune diseases, the subsequent section will delve into how dysregulation of this pathway contributes to the pathogenesis and progression of various autoimmune disorders.

1. The CD40/CD40L axis in systemic lupus erythematosus

Systemic lupus erythematosus **(**SLE) is characterised by hyperactivation of B lymphocytes leading to the production of autoantibodies, which alone or via immune complexes may be responsible for tissue damage. Given that B lymphocytes express CD40 on their surface and that CD40L/CD40 interactions are fundamental to the success of the humoral immune response, various studies have been investigating the role of the CD40L/CD40 signal in the pathophysiology of SLE since the 1990s.

Koshy et al. show that lupus patients overexpress CD40L on the surface of T lymphocytes and that the increase in expression correlates with disease activity. The authors show that this increase in CD40L expression by T lymphocytes has functional consequences, in particular through its ability to generate tumour B lymphocyte activation [31].

In addition, lupus patients have aberrant ectopic expression of CD40L by monocytes and B lymphocytes. CD40L in B lymphocytes is functional because CD40L + B lymphocytes spontaneously produce antibodies *in vitro* by a CD40L-dependent mechanism [32].

Transgenic mice for B lymphocytes expressing CD40L develop lupus-like disease with age, characterised by glomerulonephritis and spontaneous production of anti-DNA antibodies, suggesting that ectopic expression of CD40L by B lymphocytes is sufficient for autoimmunity induction [33]. Several reports have shown that patients with SLE have elevated serum levels of sCD40L which correlate with disease severity [34,35]. Western blot analysis of serum sCD40L shows that it is present in part in trimeric form and that it is functional, in particular through its ability to induce *in vitro* expression by tumour B lymphocytes of activation markers including CD95 and CD86 [35].

The CD40/CD40L axis is central to the pathophysiology of SLE, having a pro-inflammatory effect on numerous target cells. Its involvement allows the endothelial cell to interact with leukocytes [36], the mDC to activate and mature, the pDC to optimise its secretion of IFNα [[37], [38], [39]], the B lymphocyte to undergo T-dependent activation leading to proliferation, differentiation, formation of germinal centers, isotypic switching, clonal expansion of high-affinity B lymphocytes, differentiation into memory cells [6,40], the CD8^+^ T lymphocyte to optimise its cytotoxic response, and the helper T lymphocyte to activate and differentiate [41]. It should be noted that CD40L may also have other immunomodulatory interactions that are not mediated by the CD40/CD40L pair but by other, potentially synergistic, receptors. For example, CD40L is capable of stimulating platelet activation via GPIIb-IIIa or monocyte activation via integrin α5β1 [42].

In 1995, Mohan et al. described the importance of the CD40/CD40L axis in lymphocyte costimulation in lupus mouse models. The use of an anti-CD40L antibody blocked the production of T-dependent autoantibodies and the development of lupus nephritis [37]. The anti-CD40L antibody appeared to have an effect on the formation of centers, the generation of auto-antibodies and the development of memory B lymphocytes [43]. The benefits of blocking CD40L in lupus mice on auto-antibody titers, the occurrence of nephritis and overall survival have been confirmed on several occasions by subsequent studies [44,45].

In subjects with active SLE, CD40L is over-expressed by T lymphocytes, but is also ectopically expressed by B lymphocytes. This over-expression is functional, since blocking it with anti-CD40L inhibits lymphocyte production of auto-antibodies *in vitro* [32]. This ectopic expression of CD40L on B lymphocytes could be sufficient to induce autoimmunity, as suggested by the model of transgenic mice for B lymphocytes expressing CD40L; these mice being capable of developing a “lupus-like” disease with circulating auto-antibodies and glomerulonephritis with immune complex deposits [33].

In lupus patients, an increase in plasma or serum levels of sCD40L in a functional trimeric form has also been shown, and correlates with the titer of anti-DNA auto-antibodies and with SLE activity [33,35]. Platelet sCD40L potentially optimizes IFNα secretion in SLE [37], the latter itself being correlated with the disease severity [46]. The sCD40L, therefore, acts as a biomarker for SLE, and some data even suggest that an increase in sCD40L could be predictive of an imminent lupus attack [47].

Altogether, these elements suggest that the CD40L/CD40 axis could be a therapeutic target in SLE, and a therapeutic approach using anti-CD40L monoclonal antibodies (mAb) has been proposed in mouse models as well as in humans. Indeed, treatment with anti-CD40L mAb started before the onset of SLE prevents the onset of proteinuria, prolongs survival and reduces the anti-DNA antibody titer in various murine models of SLE [48,49]. In addition, administration of an anti-CD40L mAb once renal damage has set in slows disease progression, reduces proteinuria and is capable of bringing about remission in some individuals, even though it does not prevent the deposition of immune complexes in the kidney [45,50].

Similarly, two human clinical trials have been published to date, with Ruplizumab (BG9588) showing good clinical and biological efficacy. However, the trial had to be stopped due to thrombotic events [51]. Toralizumab (IDEC-131) was tested in patients with moderate lupus for 16 weeks with more mixed results [52].

2. The CD40/CD40L axis in multiple sclerosis

Multiple sclerosis (MS) is an autoimmune disease in which nerve cell sheaths in the brain and spinal cord are damaged by autoimmune mechanisms involving B and T cells [53], characterized by infiltration of lymphoid cells such as T cells (CD4^+^, CD8^+^) and B cells, myeloid cells such as DCs, monocytes and macrophages, and glial cells in the brain and spinal cord, leading to the destruction of the myelin of nerve fibers [54].

The pathophysiology of MS has been studied in an “Experimental Autoimmune Encephalomyelitis” (EAE) mouse model. Following immunisation of mice with components of myelin, immune cells infiltrated the CNS [55].

These mice have cells in the spinal cord which strongly express CD40 during the acute and relapsing phases of MS, whereas CD40L is only strongly expressed during the relapsing phase of the disease [55]. In fact, CD40L-deficient mice are protected against EAE. This is due to a defect in the maturation and activation of APCs [56]. In addition, the administration of an anti-CD40L to mice at the beginning of the induction phase of EAE prevents the development of MS [57].

Several studies have shown that blocking the interaction of CD40L with CD40 can prevent differentiation of T cells into a Th1 immune response, which can induce these cells to differentiate into a non-pathogenic Th2 immune response in the CNS [[58], [59], [60]].

CD40 is also expressed on the surface of glial cells, and its activation leads to the secretion of IL-12 and TNF, leading to cell apoptosis [[61], [62], [63]]. Other studies have reported that mice with a deletion of CD40 specifically in glial cells develop less severe EAE compared with wild-type mice [[62], [63], [64]].

One study demonstrated a positive correlation between albumin quotient (a marker of blood-brain barrier disruption) and soluble CD40L levels in the serum of MS patients [65]. In a contrary clinical study, researchers demonstrated that there was a decrease in the amounts of sCD40L in the serum of MS patients, and that the administration of interferon beta and glatiramer acetate further reduced these levels [66].

3. The CD40/CD40L axis in autoimmune and inflammatory bowel diseases

Crohn's disease and ulcerative colitis are the two main autoimmune and well decribed diseases of the intestine. Both diseases are chronic conditions characterized by inflammation of the gastrointestinal tract due to infiltration of large numbers of T and B cells and macrophages into the intestinal epithelium [67].

Patients with Crohn's disease show overexpression of CD40L on the surface of T cells and high serum levels of sCD40L [68]. CD40 is also over-expressed on the surface of microvascular endothelial cells in the intestinal mucosa [69]. Indeed, several experimental mouse models, obtained by mutagenesis of genes encoding components of the immune system such as mice deficient in the MHC class II molecule, TCR, IL-2 and others, have shown the importance of the CD40L/CD40 signaling in intestinal inflammation [70]. The administration of an anti-CD40L to mice showing the first symptoms of colitis is able to prevent the development of the disease and inhibits the infiltration of immune cells into the intestinal epithelium [71,72].

In 2005, a group studied the role of a specific CD40L antagonist, ch5D12, in the treatment of patients with Crohn's disease. Injection of this inhibitor blocks the CD40L/CD40 interaction and has shown beneficial effects. In fact, 72 % of patients showed a positive response to treatment and 22 % went into remission from the disease [73].

4. The CD40/CD40L axis in immunological thrombocytopenic purpura

Immunological thrombocytopenic purpura (ITP) is an autoimmune cytopenia associated to an antibody response directed against platelet glycoproteins. It has been shown that in ITP carriers, compared with healthy subjects, platelet CD40L expression was increased and that platelet CD40L was able to induce activation of the autoreactive B lymphocytes [74].

Sjögren's syndrome (SS) is a systemic autoimmune disease characterized by lymphocytic infiltration of the exocrine glands (justifying its name of autoimmune epithelitis) and by B lymphocyte hyperactivation (hypergammaglobulinaemia, presence of anti-SSA or anti-SSB auto-antibodies). Salivary gland epithelial cells act as antigen-presenting cells, promoting B lymphocyte activation. Indeed, there is overexpression of the CD40 pathway in the B lymphocytes of SS patients in the presence of salivary gland epithelial cells [75]. In patients with SS, CD4^+^ T lymphocytes overexpress CD40L on their surface compared with CD4^+^ T lymphocytes from healthy subjects, constituting another B lymphocyte activation pathway [76].

5. The CD40/CD40L axis in rheumatoid arthritis

Rheumatoid arthritis (RA) is a chronic inflammatory autoimmune disease featured by immune cells infiltration into the joints leading to increased production of cytokines, chemokines and MMPs. It is manifested by persistent synovitis and auto-antibodies directed against rheumatoid factor and citrullinated peptides; and subsequently leads to inflammation and joints destruction [77].

CD40L and CD40 appear to play a crucial role in the initiation and development of RA. CD40L is over-expressed on the surface of circulating T cells and in the synovium of RA patients. This aberrant expression of CD40L leads to increased production of immunoglobulins by B cells (hypergammaglobulinaemia), and increased secretion of IL-12 by DCs and synovial macrophages [[78], [79], [80]]. In addition, CD40 is also overexpressed on fibroblasts and synovial cells by the presence of TNF-α and IFN-γ [81]. Such overexpression induces an increase in the secretion of pro-inflammatory cytokines such as IL-6, IL-8, IL-15, IL-17, TNF and MCP-1, which amplifies RA-related inflammatory reactions and thus leads to joint destruction [82,83].

Binding of CD40L from activated T cells to CD40 from fibroblasts stimulates VEGF production, inducing synovial neovascularisation. Moreover, this interaction increases the expression of adhesion molecules, such as E-selectin, ICAM-1 and VCAM-1, on the surface of fibroblasts, leading to the recruitment of immune cells to the inflammation site [81]. It should also be mentioned that serum levels of sCD40L are very high in RA patients.

The role of the CD40L/CD40 dyad in the initiation of RA was demonstrated in a mouse model when an anti-CD40L monoclonal antibody was administered prior to the initiation of collagen-induced arthritis (CIA), in which the use of this antibody prevented the development of RA. Injection of this same treatment into mice that had already developed RA was unable to reverse the complications induced by CIA [84,85]. This indicates that the CD40L/CD40 pair plays a crucial role in the early pathophysiological events of RA.

## 4 Diagnostic and prognostic value of sCD40L

The generation of autoantibodies, particularly anti-native DNA, and disease activity have been linked to higher levels of sCD40L in SLE [34]. Nonetheless, indicators like C-reactive protein (CRP) and type I interferon are frequently employed to track flares and inflammation [46]. An early sign of lupus exacerbations, sCD40L increase may occur before anti-native DNA rise, according to a longitudinal research evaluating these biomarkers [47].

In multiple sclerosis (MS), sCD40L has been studied as an inflammatory biomarker. Some work shows a correlation between its serum levels and blood-brain barrier permeability, suggesting a potential role in monitoring disease progression [65]. However, biomarkers such as neurofilament light chains (NfL) are more directly linked to neurodegeneration [66].

Concerning rheumatoid arthritis (RA), elevated levels of sCD40L have been detected in affected patients, correlating with synoviocyte activation and the production of pro-inflammatory cytokines [78]However, rheumatoid factor (RF) and anti-CCP antibodies continue to be the most often used indicators for prognosis and illness diagnosis [81]. Combined analysis of these biomarkers with sCD40L could improve patient stratification according to disease activity and response to treatment [83].

## 5 Limitations of sCD40L as a biomarker

Despite its potential, there are a number of drawbacks to using sCD40L as a clinical biomarker.

1. **Assay variability**: It can be difficult to set standardized criteria since sCD40L quantification techniques (ELISA, Western blot) can produce inconsistent results [7].Moreover, the amounts recorded are influenced by the conditions of sampling and storage [3].
2. **Heterogeneous origin** of **sCD40L**: Activated platelets account for a sizable amount of circulating sCD40L, which could skew its interpretation, especially in cases with related cardiovascular disease [86].
3. **Limited specificity**: sCD40L is not exclusive to autoimmune illnesses because it is implicated in a number of inflammatory and thrombotic processes. Therefore, in order to refine its clinical usefulness, it must be evaluated in conjunction with other markers [28].

## 6 Monoclonal anti-CD40L antibodies and their alternatives

1. First-generation anti-CD40L

In 1992, the first anti-CD40L monoclonal antibody was pro-ducted, a murine IgG2 named mAb 5c8 capable of blocking B lymphocyte differentiation induced by activated CD4^+^ T lymphocytes [87]. It has been shown to prevent renal allograft rejection in primates, with good tolerability, by blocking lymphocyte costimulation synergistically with the CTLA4-Ig fusion protein [88]. In view of these promising preclinical results, several other anti-CD40L monoclonal antibodies were developed in the 2000s for clinical evaluation in humans, particularly in SLE. Ruplizumab (BG9588, hu5c8) is a humanized IgG1 anti-CD40L antibody that has been shown to reduce proteinuria, hematuria and anti-DNA antibody titer, as well as to raise C3 levels in patients with lupus glomerulonephritis. However, the study was stopped prematurely due to thrombotic events such as myocardial infarction and pulmonary embolism in treated patients [51]. Toralizumab (IDEC-131, hu24-31) is also a humanized IgG1. Having failed to raise any red flags when evaluated in 23 lupus patients in phase I trials [89], its phase II trial in SLE was conducted but failed to show superiority to placebo [52].

Two Phase I/II studies had shown modest efficacy in persistent or chronic ITP, with no thrombotic events reported, in small numbers of 31 and 20 patients [90,91]. Its exported development in other dysimmune or inflammatory pathologies was halted following thromboembolic toxicity in Crohn's disease [92,93].ABI793 is an IgG1 anti-CD40L antibody, this time fully human. Its evaluation in monkey renal transplants was associated with prolonged graft survival, but also with thromboembolic adverse events affecting various organs [94,95].

2. New-generation anti-CD40L

The thromboembolic complications of ruplizumab, tora-lizumab and ABI793, long poorly understood, led to their development being halted despite encouraging results [92]. Dapirolizumab, letolizumab, SAR441344 and VIB4920 are more recent treatments that have been developed differently, resulting in a better safety profile [[96], [97], [98], [99]] and continued development. Thromboembolic toxicity could involve the Fc fragment of the antibody: endogenous monoclonal antibody-CD40L immune complexes would be able to bind to platelets via their surface IgG receptor (Fc RIIA), thereby activating them and promoting aggregation and thrombosis [100]. This hypothesis would explain the good tolerability of dapirolizumab, letolizumab and VIB4920, which are built without an Fc fragment [98,101] or with an Fc fragment stripped of its effector functions [99]. A second hypothesis involves non-CD40 receptors for CD40L, including platelet integrin IIb 3 (GPIIb-IIIa). The interaction of this integrin with CD40L plays a role in arterial thrombus stabilization. Blocking this ligand therefore tends to destabilize the platelet plug, increasing embolic risk [102].

Among the new-generation antibodies, dapirolizumab pegol (CDP7657) is an antibody derived from a Fab fragment assembly of anti-CD40L antibodies conjugated with high-molecular-weight polyethylene glycol. Unlike the previous three antibodies, it has no Fc fragment. Its first human evaluation in healthy subjects, followed by lupus in 2015, was not associated with any thromboembolic events [103]. Its safety was confirmed in 16 lupus patients in phase I trials in 2017, with no thromboembolic adverse events [101]. Its Phase IIb evaluation on 182 patients in 2019 did not meet its primary endpoint, but showed a biological benefit of the treatment (notably on anti-DNA antibody titer) and good safety (one thromboembolic event in the treatment group, three in the placebo group) [97]. These results were deemed sufficient to initiate a placebo-controlled evaluation in lupus in 450 participants in phase III [104].

Letolizumab (BMS-986004) is a fusion protein consisting of a dimeric anti-human CD40L antibody domain linked to the Fc portion of IgG1, modified to reduce its effective functions. In a preclinical renal transplantation trial in monkeys, it showed benefit in preventing allograft rejection without causing thromboembolic events [99]. There is also an open-label Phase I/II trial underway evaluating the value of letolizumab in the prevention of postallograft hematopoietic stem cell graft-versus-host disease [105].

VIB4920 is a CD40L antagonist distinguished by its Fc-free construction, consisting of two iden-tical Tn3 proteins (CD40L-inhibiting molecules derived from human tenascin-C type III fibronectin) attached to human serum albumin. Its pharmacological study in monkeys demonstrated its ability to block CD40^−^CD40L interaction without any associated thromboembolic toxicity [98]. Phase Ib data in 57 patients with active seropositive RA demonstrated VIB4920's ability to reduce disease activity without causing thromboembolic events [96]. A proof-of-concept Phase II placebo-controlled study has been initiated in SS patients [106].

3. Prospects for nano-immunotherapy

Since the use of several anti-CD40L monoclonal antibodies, mainly first-generation ones, has been associated with thromboembolic side effects, new strategies have been developed to target a more restricted part of the CD40^−^CD40L axis. This is the case with nano-immunotherapy using TRAF-STOPs, small molecules targeting TRAFs (TNF receptor-associated factor), adaptor proteins associated with the cytoplasmic domain of the CD40 receptor, enabling CD40/CD40L signal transduction [107]. To date, only results on mouse models are available, such as the blocking of CD40-TRAF6 interaction, which has shown good tolerance and a reduction in adipocyte inflammation and hepatic steatosis in obese mice [107].

## 7 Conclusion

In summary, CD40^−^CD40L is a key component in the understanding and treatment of autoimmune diseases as well as related athérosclerosis. The latest developments in targeted therapies, such as antibiotics and nanotechnology, have opened up new and exciting avenues for investigation. However, before incorporating these approaches into clinical practices, a thorough assessment of their risks and benefits is essential. Clinical studies must be carried out to confirm the safety and efficacy of developing therapies and to investigate the potential effects of molecules already used in other fields. By doing this, we will be able to better understand the crucial role that CD40L plays in the pathophysiology of these conditions and provide patients with innovative therapeutic choices.

## CRediT authorship contribution statement

**Meryem Mabrouk:** Writing – review & editing, Writing – original draft, Validation, Conceptualization. **Hicham Wahnou:** Writing – review & editing, Visualization, Validation. **Yahye Merhi:** Writing – review & editing, Validation, Supervision. **Haissam Abou-Saleh:** Writing – review & editing, Visualization, Validation. **Fadila Guessous:** Writing – review & editing, Validation, Supervision, Conceptualization. **Younes Zaid:** Writing – review & editing, Validation, Supervision, Conceptualization.

## Declaration of competing interest

The authors declare that they have no known competing financial interests or personal relationships that could have appeared to influence the work reported in this paper.

## Data Availability

No data was used for the research described in the article.

## References

[1] Graf D., Korthäuer U., Mages H.W., Senger G., Kroczek R.A. (1992). Cloning of TRAP, a ligand for CD40 on human T cells. Eur. J. Immunol..

[2] Tay N.Q., Lee D.C.P., Chua Y.L., Prabhu N., Gascoigne N.R.J., Kemeny D.M. (2017). CD40L expression allows CD8+ T cells to promote their own expansion and differentiation through dendritic cells. Front. Immunol..

[3] Nagasawa M., Zhu Y., Isoda T., Tomizawa D., Itoh S., Kajiwara M. (2005). Analysis of serum soluble CD40 ligand (sCD40L) in the patients undergoing allogeneic stem cell transplantation: platelet is a major source of serum sCD40L. Eur. J. Haematol..

[4] André P., Nannizzi-Alaimo L., Prasad S.K., Phillips D.R. (2002). Platelet-derived CD40L: the switch-hitting player of cardiovascular disease. Circulation.

[5] Lievens D., Eijgelaar W., Biessen E., Daemen M., Lutgens E. (2009). The multi-functionality of CD40L and its receptor CD40 in atherosclerosis. Thromb. Haemost..

[6] Van Kooten C., Banchereau J. (2000). CD40-CD40 ligand. J. Leukoc. Biol..

[7] Yacoub D., Benslimane N., Al-Zoobi L., Hassan G., Nadiri A., Mourad W. (2013). CD154 is released from T-cells by a disintegrin and metalloproteinase domain-containing protein 10 (ADAM10) and ADAM17 in a CD40 protein-dependent manner. J. Biol. Chem..

[8] Choi W.‐S., Jeon O.‐H., Kim D.‐S. (2010). CD40 ligand shedding is regulated by interaction between matrix metalloproteinase‐2 and platelet integrin αIIbβ3. J. Thromb. Haemostasis.

[9] Elgueta R., Benson M.J., De Vries V.C., Wasiuk A., Guo Y., Noelle R.J. (2009). Molecular mechanism and function of CD40/CD40L engagement in the immune system. Immunol. Rev..

[10] van Kooten C., Banchereau J. (1997). Functions of CD40 on B cells, dendritic cells and other cells. Curr. Opin. Immunol..

[11] Wagner A.H., Güldenzoph B., Lienenlüke B., Hecker M. (2004). CD154/CD40-mediated expression of CD154 in endothelial cells: consequences for endothelial cell-monocyte interaction. Arterioscler. Thromb. Vasc. Biol..

[12] Ma Y.-C., Lee W.-I., Shyur S.-D., Lin S.-C., Huang L.-H., Wu J.-Y. (2005). De novo mutation causing X-linked hyper-IgM syndrome: a family study in Taiwan. Asian Pac. J. Allergy Immunol..

[13] Chatzigeorgiou A., Lyberi M., Chatzilymperis G., Nezos A., Kamper E. (2009). CD40/CD40L signaling and its implication in health and disease. Biofactors.

[14] Karnell J.L., Rieder S.A., Ettinger R., Kolbeck R. (2019). Targeting the CD40-CD40L pathway in autoimmune diseases: humoral immunity and beyond. Adv. Drug Deliv. Rev..

[15] Kawai T., Andrews D., Colvin R.B., Sachs D.H., Cosimi A.B. (2000). Thromboembolic complications after treatment with monoclonal antibody against CD40 ligand. Nat. Med..

[16] Chittasupho C., Siahaan T.J., Vines C.M., Berkland C. (2011). Autoimmune therapies targeting costimulation and emerging trends in multivalent therapeutics. Ther. Deliv..

[17] Gordon J., Pound J.D. (2000). Fortifying B cells with CD154: an engaging tale of many hues. Immunology.

[18] Korthäuer U., Graf D., Mages H.W., Brière F., Padayachee M., Malcolm S. (1993). Defective expression of T-cell CD40 ligand causes X-linked immunodeficiency with hyper-IgM. Nature.

[19] Randall T.D., Heath A.W., Santos-Argumedo L., Howard M.C., Weissman I.L., Lund F.E. (1998). Arrest of B lymphocyte terminal differentiation by CD40 signaling: mechanism for lack of antibody-secreting cells in germinal centers. Immunity.

[20] Grewal I.S., Flavell R.A. (1998). CD40 and CD154 in cell-mediated immunity. Annu. Rev. Immunol..

[21] Janeway C.A., Bottomly K. (1994). Signals and signs for lymphocyte responses. Cell.

[22] Caux C., Massacrier C., Vanbervliet B., Dubois B., Van Kooten C., Durand I. (1994). Activation of human dendritic cells through CD40 cross-linking. J. Exp. Med..

[23] Del Cornò M., Gauzzi M.C., Penna G., Belardelli F., Adorini L., Gessani S. (2005). Human immunodeficiency virus type 1 gp120 and other activation stimuli are highly effective in triggering alpha interferon and CC chemokine production in circulating plasmacytoid but not myeloid dendritic cells. J. Virol..

[24] Cella M., Scheidegger D., Palmer-Lehmann K., Lane P., Lanzavecchia A., Alber G. (1996). Ligation of CD40 on dendritic cells triggers production of high levels of interleukin-12 and enhances T cell stimulatory capacity: T-T help via APC activation. J. Exp. Med..

[25] Grewal I.S., Xu J., Flavell R.A. (1995). Impairment of antigen-specific T-cell priming in mice lacking CD40 ligand. Nature.

[26] Buhlmann J.E., Gonzalez M., Ginther B., Panoskaltsis-Mortari A., Blazar B.R., Greiner D.L. (1999). Cutting edge: sustained expansion of CD8+ T cells requires CD154 expression by Th cells in acute graft versus host disease. J. Immunol..

[27] Schoenberger S.P., Toes R.E., van der Voort E.I., Offringa R., Melief C.J. (1998). T-cell help for cytotoxic T lymphocytes is mediated by CD40-CD40L interactions. Nature.

[28] Gerdes N., Seijkens T., Lievens D., Kuijpers M.J.E., Winkels H., Projahn D. (2016). Platelet CD40 exacerbates atherosclerosis by transcellular activation of endothelial cells and leukocytes. Arterioscler. Thromb. Vasc. Biol..

[29] Zuchtriegel G., Uhl B., Puhr-Westerheide D., Pörnbacher M., Lauber K., Krombach F. (2016). Platelets guide leukocytes to their sites of extravasation. PLoS Biol..

[30] Seijkens T.T.P., van Tiel C.M., Kusters P.J.H., Atzler D., Soehnlein O., Zarzycka B. (2018). Targeting CD40-induced TRAF6 signaling in macrophages reduces atherosclerosis. J. Am. Coll. Cardiol..

[31] Koshy M., Berger D., Crow M.K. (1996). Increased expression of CD40 ligand on systemic lupus erythematosus lymphocytes. J. Clin. Investig..

[32] Desai-Mehta A., Lu L., Ramsey-Goldman R., Datta S.K. (1996). Hyperexpression of CD40 ligand by B and T cells in human lupus and its role in pathogenic autoantibody production. J. Clin. Investig..

[33] Higuchi T., Aiba Y., Nomura T., Matsuda J., Mochida K., Suzuki M. (2002). Cutting edge: ectopic expression of CD40 ligand on B cells induces lupus-like autoimmune disease. J. Immunol..

[34] Kato K., Santana-Sahagún E., Rassenti L.Z., Weisman M.H., Tamura N., Kobayashi S. (1999). The soluble CD40 ligand sCD154 in systemic lupus erythematosus. J. Clin. Investig..

[35] Vakkalanka R.K., Woo C., Kirou K.A., Koshy M., Berger D., Crow M.K. (1999). Elevated levels and functional capacity of soluble CD40 ligand in systemic lupus erythematosus sera. Arthritis Rheum..

[36] Hendrickson W.A., Ward K.B. (1975). Atomic models for the polypeptide backbones of myohemerythrin and hemerythrin. Biochem. Biophys. Res. Commun..

[37] Makar A.B., McMartin K.E., Palese M., Tephly T.R. (1975). Formate assay in body fluids: application in methanol poisoning. Biochem. Med..

[38] Nomura S., Fujita S., Nakanishi T., Yokoi T., Shimamoto K., Miyamoto R. (2012). Platelet-derived microparticles cause CD154-dependent activation of dendritic cells. Platelets.

[39] Kaneider N.C., Kaser A., Tilg H., Ricevuti G., Wiedermann C.J. (2003). CD40 ligand-dependent maturation of human monocyte-derived dendritic cells by activated platelets. Int. J. Immunopathol. Pharmacol..

[40] Delmas Y., Viallard J.-F., Villeneuve J., Grosset C., Pasquet J.-M., Déchanet-Merville J. (2005). [Platelet-associated CD154: a new interface in haemostasis and in the inflammatory reaction]. Med. Sci..

[41] Mackey M.F., Barth R.J., Noelle R.J. (1998). The role of CD40/CD154 interactions in the priming, differentiation, and effector function of helper and cytotoxic T cells. J. Leukoc. Biol..

[42] Alturaihi H., Hassan G.S., Al-Zoobi L., Salti S., Darif Y., Yacoub D. (2015). Interaction of CD154 with different receptors and its role in bidirectional signals. Eur. J. Immunol..

[43] Foy T.M., Laman J.D., Ledbetter J.A., Aruffo A., Claassen E., Noelle R.J. (1994). gp39-CD40 interactions are essential for germinal center formation and the development of B cell memory. J. Exp. Med..

[44] Early G.S., Zhao W., Burns C.M. (1996). Anti-CD40 ligand antibody treatment prevents the development of lupus-like nephritis in a subset of New Zealand black x New Zealand white mice. Response correlates with the absence of an anti-antibody response. J. Immunol..

[45] Kalled S.L., Cutler A.H., Datta S.K., Thomas D.W. (1998). Anti-CD40 ligand antibody treatment of SNF1 mice with established nephritis: preservation of kidney function. J. Immunol..

[46] Mathian A., Mouries-Martin S., Dorgham K., Devilliers H., Barnabei L., Ben Salah E. (2019). Monitoring disease activity in systemic lupus erythematosus with single-molecule array digital enzyme-linked immunosorbent assay quantification of serum interferon-α. Arthritis Rheumatol..

[47] Munroe M.E., Vista E.S., Guthridge J.M., Thompson L.F., Merrill J.T., James J.A. (2014). Proinflammatory adaptive cytokine and shed tumor necrosis factor receptor levels are elevated preceding systemic lupus erythematosus disease flare. Arthritis Rheumatol..

[48] Mohan C., Shi Y., Laman J.D., Datta S.K. (1995). Interaction between CD40 and its ligand gp39 in the development of murine lupus nephritis. J. Immunol..

[49] Wang X., Huang W., Schiffer L.E., Mihara M., Akkerman A., Hiromatsu K. (2003). Effects of anti-CD154 treatment on B cells in murine systemic lupus erythematosus. Arthritis Rheum..

[50] Quezada S.A., Eckert M., Adeyi O.A., Schned A.R., Noelle R.J., Burns C.M. (2003). Distinct mechanisms of action of anti-CD154 in early versus late treatment of murine lupus nephritis. Arthritis Rheum..

[51] Boumpas D.T., Furie R., Manzi S., Illei G.G., Wallace D.J., Balow J.E. (2003). A short course of BG9588 (anti-CD40 ligand antibody) improves serologic activity and decreases hematuria in patients with proliferative lupus glomerulonephritis. Arthritis Rheum..

[52] Kalunian K.C., Davis J.C., Merrill J.T., Totoritis M.C., Wofsy D., IDEC-131 Lupus Study Group (2002). Treatment of systemic lupus erythematosus by inhibition of T cell costimulation with anti-CD154: a randomized, double-blind, placebo-controlled trial. Arthritis Rheum..

[53] Tintore M., Vidal-Jordana A., Sastre-Garriga J. (2019). Treatment of multiple sclerosis - success from bench to bedside. Nat. Rev. Neurol..

[54] Sospedra M., Martin R. (2005). Immunology of multiple sclerosis. Annu. Rev. Immunol..

[55] Issazadeh S., Navikas V., Schaub M., Sayegh M., Khoury S. (1998). Kinetics of expression of costimulatory molecules and their ligands in murine relapsing experimental autoimmune encephalomyelitis in vivo. J. Immunol..

[56] Grewal I.S., Foellmer H.G., Grewal K.D., Xu J., Hardardottir F., Baron J.L. (1996). Requirement for CD40 ligand in costimulation induction, T cell activation, and experimental allergic encephalomyelitis. Science.

[57] Gerritse K., Laman J.D., Noelle R.J., Aruffo A., Ledbetter J.A., Boersma W.J. (1996). CD40-CD40 ligand interactions in experimental allergic encephalomyelitis and multiple sclerosis. Proc. Natl. Acad. Sci. U. S. A..

[58] Howard L.M., Miga A.J., Vanderlugt C.L., Dal Canto M.C., Laman J.D., Noelle R.J. (1999). Mechanisms of immunotherapeutic intervention by anti-CD40L (CD154) antibody in an animal model of multiple sclerosis. J. Clin. Investig..

[59] Howard L.M., Miller S.D. (2001). Autoimmune intervention by CD154 blockade prevents T cell retention and effector function in the target organ. J. Immunol..

[60] Samoilova E.B., Horton J.L., Zhang H., Chen Y. (1997). CD40L blockade prevents autoimmune encephalomyelitis and hampers TH1 but not TH2 pathway of T cell differentiation. J. Mol. Med. (Berl.).

[61] Becher B., Blain M., Antel J.P. (2000). CD40 engagement stimulates IL-12 p70 production by human microglial cells: basis for Th1 polarization in the CNS. J. Neuroimmunol..

[62] Ponomarev E.D., Shriver L.P., Dittel B.N. (2006). CD40 expression by microglial cells is required for their completion of a two-step activation process during central nervous system autoimmune inflammation. J. Immunol..

[63] Tan J., Town T., Paris D., Placzek A., Parker T., Crawford F. (1999). Activation of microglial cells by the CD40 pathway: relevance to multiple sclerosis. J. Neuroimmunol..

[64] Becher B., Durell B.G., Miga A.V., Hickey W.F., Noelle R.J. (2001). The clinical course of experimental autoimmune encephalomyelitis and inflammation is controlled by the expression of CD40 within the central nervous system. J. Exp. Med..

[65] Masuda H., Mori M., Uchida T., Uzawa A., Ohtani R., Kuwabara S. (2017). Soluble CD40 ligand contributes to blood-brain barrier breakdown and central nervous system inflammation in multiple sclerosis and neuromyelitis optica spectrum disorder. J. Neuroimmunol..

[66] de J Guerrero-García J., Rojas-Mayorquín A.E., Valle Y., Padilla-Gutiérrez J.R., Castañeda-Moreno V.A., Mireles-Ramírez M.A. (2018). Decreased serum levels of sCD40L and IL-31 correlate in treated patients with Relapsing-Remitting Multiple Sclerosis. Immunobiology.

[67] Cho J.H. (2008). Inflammatory bowel disease: genetic and epidemiologic considerations. World J. Gastroenterol..

[68] Anka Idrissi D., Senhaji N., Aouiss A., Khalki L., Tijani Y., Zaid N. (2021). IL-1 and CD40/CD40L platelet complex: elements of induction of Crohn's disease and new therapeutic targets. Arch Pharm. Res. (Seoul).

[69] Danese S., Sans M., Scaldaferri F., Sgambato A., Rutella S., Cittadini A. (2006). TNF-alpha blockade down-regulates the CD40/CD40L pathway in the mucosal microcirculation: a novel anti-inflammatory mechanism of infliximab in Crohn's disease. J. Immunol..

[70] Senhaji N., Kojok K., Darif Y., Fadainia C., Zaid Y. (2015). The contribution of CD40/cd40l Axis in inflammatory bowel disease: an update. Front. Immunol..

[71] Liu Z., Geboes K., Colpaert S., Overbergh L., Mathieu C., Heremans H. (2000). Prevention of experimental colitis in SCID mice reconstituted with CD45RBhigh CD4+ T cells by blocking the CD40-CD154 interactions. J. Immunol..

[72] Stuber E., Strober W., Neurath M. (1996). Blocking the CD40L-CD40 interaction in vivo specifically prevents the priming of T helper 1 cells through the inhibition of interleukin 12 secretion. J. Exp. Med..

[73] Kasran A., Boon L., Wortel C.H., Hogezand R.A., Schreiber S., Goldin E. (2005). Safety and tolerability of antagonist anti-human CD40 Mab ch5D12 in patients with moderate to severe Crohn's disease. Aliment. Pharmacol. Ther..

[74] Solanilla A., Pasquet J.-M., Viallard J.-F., Contin C., Grosset C., Déchanet-Merville J. (2005). Platelet-associated CD154 in immune thrombocytopenic purpura. Blood.

[75] Rivière E., Pascaud J., Tchitchek N., Boudaoud S., Paoletti A., Ly B. (2020). Salivary gland epithelial cells from patients with Sjögren’s syndrome induce B-lymphocyte survival and activation. Ann. Rheum. Dis..

[76] Belkhir R., Gestermann N., Koutero M., Seror R., Tost J., Mariette X. (2014). Upregulation of membrane‐bound CD 40L on CD 4 ^+^ T cells in women with primary sjögren’s syndrome. Scand. J. Immunol..

[77] Noss E.H., Brenner M.B. (2008). The role and therapeutic implications of fibroblast-like synoviocytes in inflammation and cartilage erosion in rheumatoid arthritis. Immunol. Rev..

[78] Harigai M., Hara M., Nakazawa S., Fukasawa C., Ohta S., Sugiura T. (1999). Ligation of CD40 induced tumor necrosis factor-alpha in rheumatoid arthritis: a novel mechanism of activation of synoviocytes. J. Rheumatol..

[79] MacDonald K.P., Nishioka Y., Lipsky P.E., Thomas R. (1997). Functional CD40 ligand is expressed by T cells in rheumatoid arthritis. J. Clin. Investig..

[80] Kitagawa M., Mitsui H., Nakamura H., Yoshino S., Miyakawa S., Ochiai N. (1999). Differential regulation of rheumatoid synovial cell interleukin-12 production by tumor necrosis factor alpha and CD40 signals. Arthritis Rheum..

[81] Peters A.L., Stunz L.L., Bishop G.A. (2009). CD40 and autoimmunity: the dark side of a great activator. Semin. Immunol..

[82] Cho M.-L., Yoon C.-H., Hwang S.-Y., Park M.-K., Min S.-Y., Lee S.-H. (2004). Effector function of type II collagen-stimulated T cells from rheumatoid arthritis patients: cross-talk between T cells and synovial fibroblasts. Arthritis Rheum..

[83] Min D.-J., Cho M.-L., Lee S.-H., Min S.-Y., Kim W.-U., Min J.-K. (2004). Augmented production of chemokines by the interaction of type II collagen-reactive T cells with rheumatoid synovial fibroblasts. Arthritis Rheum..

[84] Durie F.H., Fava R.A., Foy T.M., Aruffo A., Ledbetter J.A., Noelle R.J. (1993). Prevention of collagen-induced arthritis with an antibody to gp39, the ligand for CD40. Science.

[85] Kyburz D., Carson D.A., Corr M. (2000). The role of CD40 ligand and tumor necrosis factor alpha signaling in the transgenic K/BxN mouse model of rheumatoid arthritis. Arthritis Rheum..

[86] André P., Nannizzi-Alaimo L., Prasad S.K., Phillips D.R. (2002). Platelet-derived CD40L: the switch-hitting player of cardiovascular disease. Circulation.

[87] Lederman S., Yellin M.J., Krichevsky A., Belko J., Lee J.J., Chess L. (1992). Identification of a novel surface protein on activated CD4+ T cells that induces contact-dependent B cell differentiation (help). J. Exp. Med..

[88] Kirk A.D., Harlan D.M., Armstrong N.N., Davis T.A., Dong Y., Gray G.S. (1997). CTLA4-Ig and anti-CD40 ligand prevent renal allograft rejection in primates. Proc. Natl. Acad. Sci. U. S. A..

[89] Davis J.C., Totoritis M.C., Rosenberg J., Sklenar T.A., Wofsy D. (2001). Phase I clinical trial of a monoclonal antibody against CD40-ligand (IDEC-131) in patients with systemic lupus erythematosus. J. Rheumatol..

[90] Kuwana M. (2003). Effect of a single injection of humanized anti-CD154 monoclonal antibody on the platelet-specific autoimmune response in patients with immune thrombocytopenic purpura. Blood.

[91] Patel V.L., Schwartz J., Bussel J.B. (2008). The effect of anti-CD40 ligand in immune thrombocytopenic purpura. Br. J. Haematol..

[92] Law C.-L., Grewal I.S. (2009). Therapeutic interventions targeting CD40L (CD154) and CD40: the opportunities and challenges. Adv. Exp. Med. Biol..

[93] Dumont F.J. (2002). IDEC-131. IDEC/Eisai. Curr. Opin. Invest. Drugs.

[94] Kanmaz T., Fechner J.H., Torrealba J., Kim H.T., Dong Y., Oberley T.D. (2004). Monotherapy with the novel human anti-CD154 monoclonal antibody ABI793 in rhesus monkey renal transplantation model1. Transplantation.

[95] Schuler W., Bigaud M., Brinkmann V., Di Padova F., Geisse S., Gram H. (2004). Efficacy and safety of ABI793, a novel human anti-human CD154 monoclonal antibody. cynomolgus monkey renal allotransplantation1: Transplantation.

[96] Karnell J.L., Albulescu M., Drabic S., Wang L., Moate R., Baca M. (2019). A CD40L-targeting protein reduces autoantibodies and improves disease activity in patients with autoimmunity. Sci. Transl. Med..

[97] Furie R., Bruce I.N., Dörner T., Leon M.G., Leszczynski P., Urowitz M.B. (2019). FRI0195 efficacy and safety of dapirolizumab pegol (dzp) in patients with moderately to severely active systemic lupus erythematosus (SLE): a randomised, placebo (PBO)-CONTROLLED study. Ann. Rheum. Dis..

[98] Nicholson S.M., Casey K.A., Gunsior M., Drabic S., Iverson W., Cook H. (2020). The enhanced immunopharmacology of VIB4920, a novel Tn3 fusion protein and CD40L antagonist, and assessment of its safety profile in cynomolgus monkeys. Br. J. Pharmacol..

[99] Kim S.C., Wakwe W., Higginbotham L.B., Mathews D.V., Breeden C.P., Stephenson A.C. (2017). Fc-silent anti-cd154 domain antibody effectively prevents nonhuman primate renal allograft rejection. Am. J. Transplant..

[100] Langer F., Ingersoll S.B., Amirkhosravi A., Meyer T., Siddiqui F.A., Ahmad S. (2005). The role of CD40 in CD40L- and antibody-mediated platelet activation. Thromb. Haemost..

[101] Chamberlain C., Colman P.J., Ranger A.M., Burkly L.C., Johnston G.I., Otoul C. (2017). Repeated administration of dapirolizumab pegol in a randomised phase I study is well tolerated and accompanied by improvements in several composite measures of systemic lupus erythematosus disease activity and changes in whole blood transcriptomic profiles. Ann. Rheum. Dis..

[102] André P., Prasad K.S.S., Denis C.V., He M., Papalia J.M., Hynes R.O. (2002). CD40L stabilizes arterial thrombi by a beta3 integrin--dependent mechanism. Nat. Med..

[103] Tocoian A., Buchan P., Kirby H., Soranson J., Zamacona M., Walley R. (2015). First-in-human trial of the safety, pharmacokinetics and immunogenicity of a PEGylated anti-CD40L antibody fragment (CDP7657) in healthy individuals and patients with systemic lupus erythematosus. Lupus.

[104] Clowse M, Isenberg D, Merrill J, Dörner T, Petri M, Vital E, et al. Dapirolizumab pegol demonstrated significant improvement in systemic lupus erythematosus disease activityEfficacy and Safety Results of a Phase 3 Trial n.D.

[105] Khimani F., Ali H., Kim J., Cubitt C., Zhang S., Elmariah H. (2025). CD40 ligand blockade for prevention of graft-versus-host disease. JCO Oncology Advances.

[106] Bose K.S., Sarma R.H. (1975). Delineation of the intimate details of the backbone conformation of pyridine nucleotide coenzymes in aqueous solution. Biochem. Biophys. Res. Commun..

[107] Chatzigeorgiou A., Seijkens T., Zarzycka B., Engel D., Poggi M., van den Berg S. (2014). Blocking CD40-TRAF6 signaling is a therapeutic target in obesity-associated insulin resistance. Proc. Natl. Acad. Sci. U. S. A..

